# Managing risk by the weakest link: Are we training effectively in the heat?

**DOI:** 10.1186/2046-7648-4-S1-A101

**Published:** 2015-09-14

**Authors:** Andrew P Hunt, Joanne N Caldwell, Daniel C Billing, Mark J Patterson

**Affiliations:** 1 Land Division, Defence Science and Technology Organisation, Melbourne, Australia; 2 Centre for Human and Applied Physiology, University of Wollongong, Wollongong, Australia

## Introduction

The Australian Army Working in Heat policy dictates limits to physical work duration to minimise the risk of heat casualties. However, commanders suggest that strict adherence to the policy prevents the majority of personnel from engaging in physical training to the limits that are physiologically tolerable in the heat. Therefore, this study examined the heat strain of personnel performing a common military activity (forced march) in environmental conditions close to the policy limits. The aim was to determine the proportion of personnel at risk of becoming heat casualties.

## Methods

Thirty-seven Royal Australian Infantry soldiers volunteered to participate in a march of up to 10 km. Participants wore a standard combat uniform and boots while carrying 40 kg of military equipment. The participants commenced the march in a rested thermoneutral state (5:30 am) after having ingested a temperature sensor at least 7 h prior. The pace of the march was guided by timing feedback at 2.5 km intervals to maintain a pace of ~5.5 km.h^-1^. The wet-bulb globe temperature (WBGT) rose through the range 21-26 °C over the course of the march, averaging 23.1(1.8) °C, which spanned the policy limit (22 °C) for this combination of protective clothing attire and work intensity. Participants rated the severity of environmental symptoms pertinent to work in the heat after the march [1].

## Results

**Figure 1:**
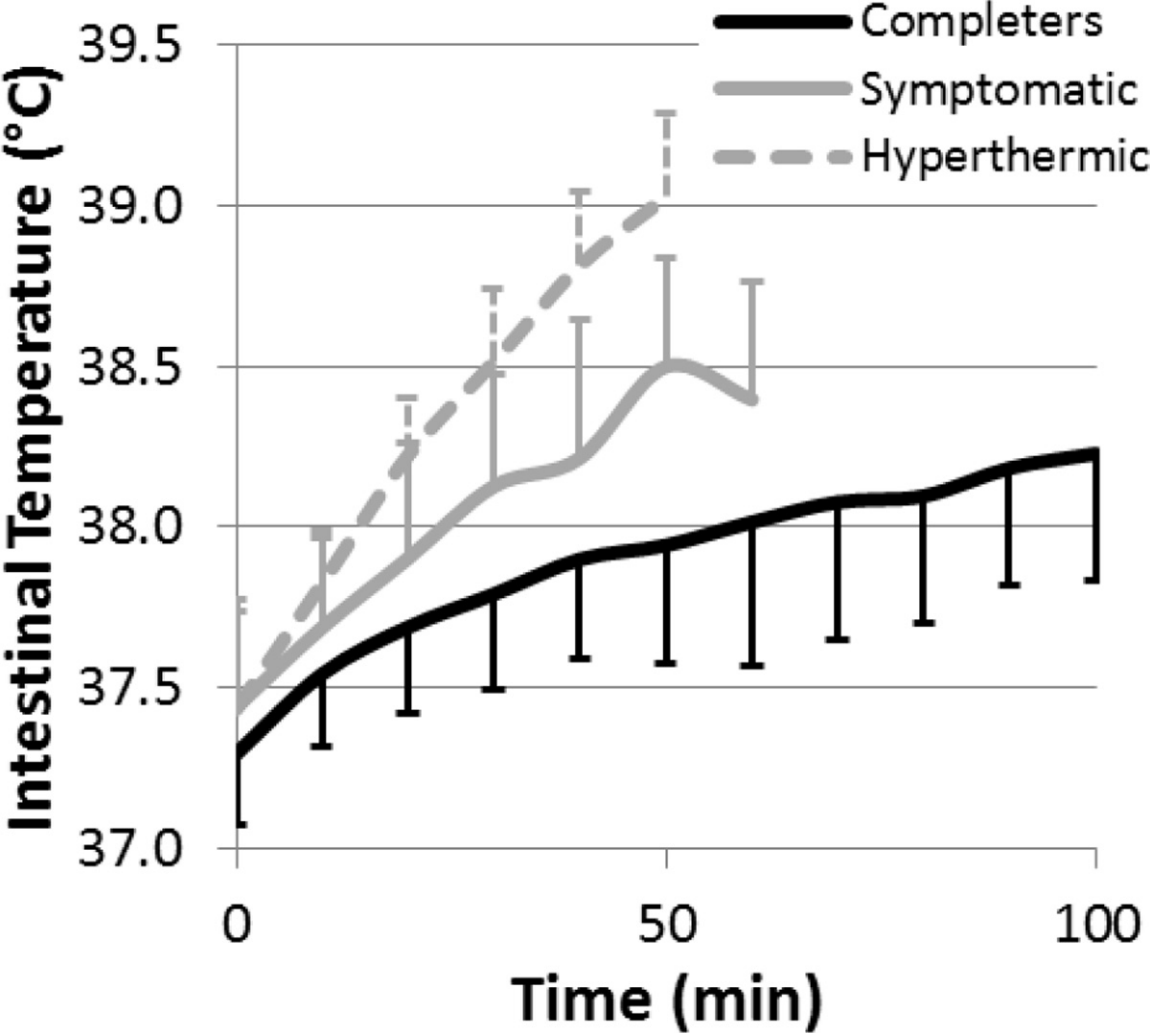
**Intestinal temperature during the march for the completers, symptomatic, and hyperthermic**.

Twenty-three (62%) participants completed the march in 107(6.4) min (Completers). Nine (24%) participants were symptomatic for heat exhaustion and withdrew from the march after 71.6(10.1) min (Symptomatic). Five (14%) participants were removed from the march when their intestinal temperature rose above 39.0 °C (Hyperthermic; Figure 1), which occurred after 58.4(4.5) min. The Symptomatic group reported significantly higher sum of environmental symptoms severity: 28(12); compared to the Completers: 12(8), *P *= 0.06; and the Hyperthermic: 13(10), *P *= 0.029.

## Discussion

Working in the heat, up to and above the recommended limitations, revealed that the policy limits coincide with a proportion of personnel at risk of becoming heat casualties. However, the findings also show that the majority of personnel did not experience excessive heat strain that would endanger health or impair performance.

## Conclusion

The current Australian Army Working in Heat Policy is an effective risk management strategy to highlight work activities which will expose a portion of personnel to excessive heat strain. However, further research is required to better inform military commanders on strategies to safely and effectively train all personnel for the rigours of physically demanding work in hot environments.
